# S-EMG signal compression based on domain transformation and spectral shape dynamic bit allocation

**DOI:** 10.1186/1475-925X-13-22

**Published:** 2014-02-27

**Authors:** Marcel Henrique Trabuco, Marcus Vinícius Chaffim Costa, Francisco Assis de Oliveira Nascimento

**Affiliations:** 1 Group of Digital Signal Processing, Department of Electrical Engineering, University of Brasília, Brasília, DF, Brazil; 2 Electronic Engineering, University of Brasília at Gama, Gama, DF, Brazil

## Abstract

**Background:**

Surface electromyographic (S-EMG) signal processing has been emerging in the past few years due to its non-invasive assessment of muscle function and structure and because of the fast growing rate of digital technology which brings about new solutions and applications. Factors such as sampling rate, quantization word length, number of channels and experiment duration can lead to a potentially large volume of data. Efficient transmission and/or storage of S-EMG signals are actually a research issue. That is the aim of this work.

**Methods:**

This paper presents an algorithm for the data compression of surface electromyographic (S-EMG) signals recorded during isometric contractions protocol and during dynamic experimental protocols such as the cycling activity. The proposed algorithm is based on discrete wavelet transform to proceed spectral decomposition and de-correlation, on a dynamic bit allocation procedure to code the wavelets transformed coefficients, and on an entropy coding to minimize the remaining redundancy and to pack all data. The bit allocation scheme is based on mathematical decreasing spectral shape models, which indicates a shorter digital word length to code high frequency wavelets transformed coefficients. Four bit allocation spectral shape methods were implemented and compared: decreasing exponential spectral shape, decreasing linear spectral shape, decreasing square-root spectral shape and rotated hyperbolic tangent spectral shape.

**Results:**

The proposed method is demonstrated and evaluated for an isometric protocol and for a dynamic protocol using a real S-EMG signal data bank. Objective performance evaluations metrics are presented. In addition, comparisons with other encoders proposed in scientific literature are shown.

**Conclusions:**

The decreasing bit allocation shape applied to the quantized wavelet coefficients combined with arithmetic coding results is an efficient procedure. The performance comparisons of the proposed S-EMG data compression algorithm with the established techniques found in scientific literature have shown promising results.

## Background

Surface Electromyographic Signals (S-EMG) have attracted greater attention from areas that deal with physiotherapy, biomechanics, sports and orthopedic medicine. Two main reasons may be reported upon: 1) S-EMG allows accessing the muscular structure and function through a non-invasive process and; 2) technologies associated with the acquisition and treatment of signals have reached a level where studies and applications based on surface electromyography have become viable.

Applications in biomechanics associated with cutting-edge sports activities may have experimental protocols that last more than fifteen minutes. Constructing an S-EMG signal data bank is important in that it makes it possible to develop research aimed at understanding physiological processes, establishing new objective parameters for analysis (for example, muscle fatigue indicators) and proposing new protocols for training in order to achieve the level of quality desired in a shorter time and without causing injuries to athletes. Storing great quantities of digitized S-EMG signals especially, those whose protocols have long durations, brings about the need for large amounts of mass memory for storing information of interest. Storage also requires an extended time for allocating channels of communication for transferring the experiment data carried out in the field (for example, experimental protocols in a cycling velodrome). Scanning an S-EMG signal involves sampling the signal which generally varies between 1 kHz and 4 kHz and quantization with a 2 byte digital word length per sample (the majority of electromyograms use 12 bit to 16 bit A/D converters). Coding with fewer bits for representing the S-EMG signal waveforms, while avoiding any significant degradation to the original information, constitutes the goal of this work.

Many different approaches for S-EMG signal compression may be found in scientific literature involving parametric coding [1,2] and waveforms coding [2,3]. Carotti *et al.*[1] proposed a linear prediction technique dedicated to S-EMG which has the advantage of low computational cost and a great gain in compression. Nevertheless, although this process is able to make a good approximation of the spectral envelope of the signal magnitude, phase information cannot be preserved. This leads to degradation of the reconstructed waveform when compared with the original. An improvement in performance may be obtained by using algorithms such as ACELP–Algebraic Code-Excited Linear Prediction [4]. On the other hand, the waveform coders have a significantly greater performance than the linear prediction when compared to the compression gain versus the signal to noise ratio. In consequence, there is also a substantial increase in the computational complexity. Norris and Lovely [5] studied a compression technique based on ADPCM–Adaptive Differential Pulse Code Modulation. Wellig *et al.*[6] and Norris *et al.*[7] investigated techniques based on a single-tree algorithm that searches for the best bases from the library of wavelet packet bases and modified EZW (Embedded Zero-tree Wavelets). Other techniques based on wavelet transforms seeking optimization of the bases for S-EMG representation may be found in scientific literature [8,9]. Techniques based on mixed or vector quantization are also present in scientific literature [10-12]. An approach involving learning about the S-EMG spectral shape with the objective of optimizing dynamic bit allocation in the quantization of wavelet transform coefficients is proposed in Berger *et al*. [13,14]. The S-EMG signal compression technique based on recurrent patterns, proposed by Filho *et al.*[15], performed excellently (compression gain versus signal to noise ratio) in isometrics experimental protocols. Nonetheless, the computational cost is greater than the techniques that utilize wavelet transforms and it is dependent on the size and spectral behavior of the signal data bank.

Multidimensional techniques may also be found in scientific literature, Carotti *et al.*[16-18] which have approaches based on linear prediction for application in multichannel electromyography. In Costa *et al.*[19-21] an approach is proposed where the S-EMG signal is segmented and juxtaposed in order to construct a two-dimensional signal (*NxM* sample matrix). In the second step, the lines which have greater correlation with each other are shifted so that they are immediately placed one after the other (the indices of their original positions are kept as side information for the decoding process). Finally, techniques for coding two-dimensional signals are applied. Other 2D techniques that use transforms and fractals appear in recent publications [22-24]. In Salman *et al.*[25] and Dixon *et al.*[26] compressed sensing is applied to S-EMG compression.

This paper presents a technique based on the wavelet transform that proposes dynamic bit allocation derived from spectral shape models and arithmetic coding applied to the data resulting from the lossy compression process. In dynamic bit allocation the spectral signal in the wavelet domain is segmented into sub-bands. The transform coefficients in each sub-band are quantized according to the spectral shape model. This produces a sequence of symbols suitable for the entropy coding utilized. In the dynamic bit allocation strategy an approximation of the behavior of the energy magnitude contour in the wavelet transform domain was sought. Performance evaluation results along with a real signal bank source are presented here. The technique shows itself to be very efficient in respect to performance evaluation metrics when compared to the variety of techniques reported in the references.

## Methods

### Proposed S-EMG compression algorithm

In the S-EMG coding process, firstly, the signal is segmented into windows. The Discrete Wavelet Transform (DWT) is applied at each window leading to a vector of transform coefficients. The signal spectrum in the wavelet domain is also segmented into sub-bands; the coefficients are quantized with a bit amount as indicated by the respective sub-band spectral shape parameter. In the next step, a lossless compression technique is applied to the set of quantized coefficients. The data are finally packed and are ready for transmission and/or storage.

In the quantization process, the amount of bits to be allocated to the transform coefficients belonging to a specific sub-band is provided by a spectral shape model curve decreasing in energy, which aims to estimate the spectral behavior of the transform coefficient vector. The efficiency of the method depends on the reasonability of the shape proposed in relation to the spectral characteristics of the signal that is being coded. The purpose of using the spectral shape model decreasing curve is to provide a better efficiency coding, since the higher energy transformed coefficients, namely those which carry a greater amount of signal information are quantized with a large number of bits, causing them to be better represented and more accurately reconstructed in the decoding process. The output of the quantization process is the input of the entropy coding used here with the goal of reducing the redundancies that still remain in the data.

**Figure 1:**
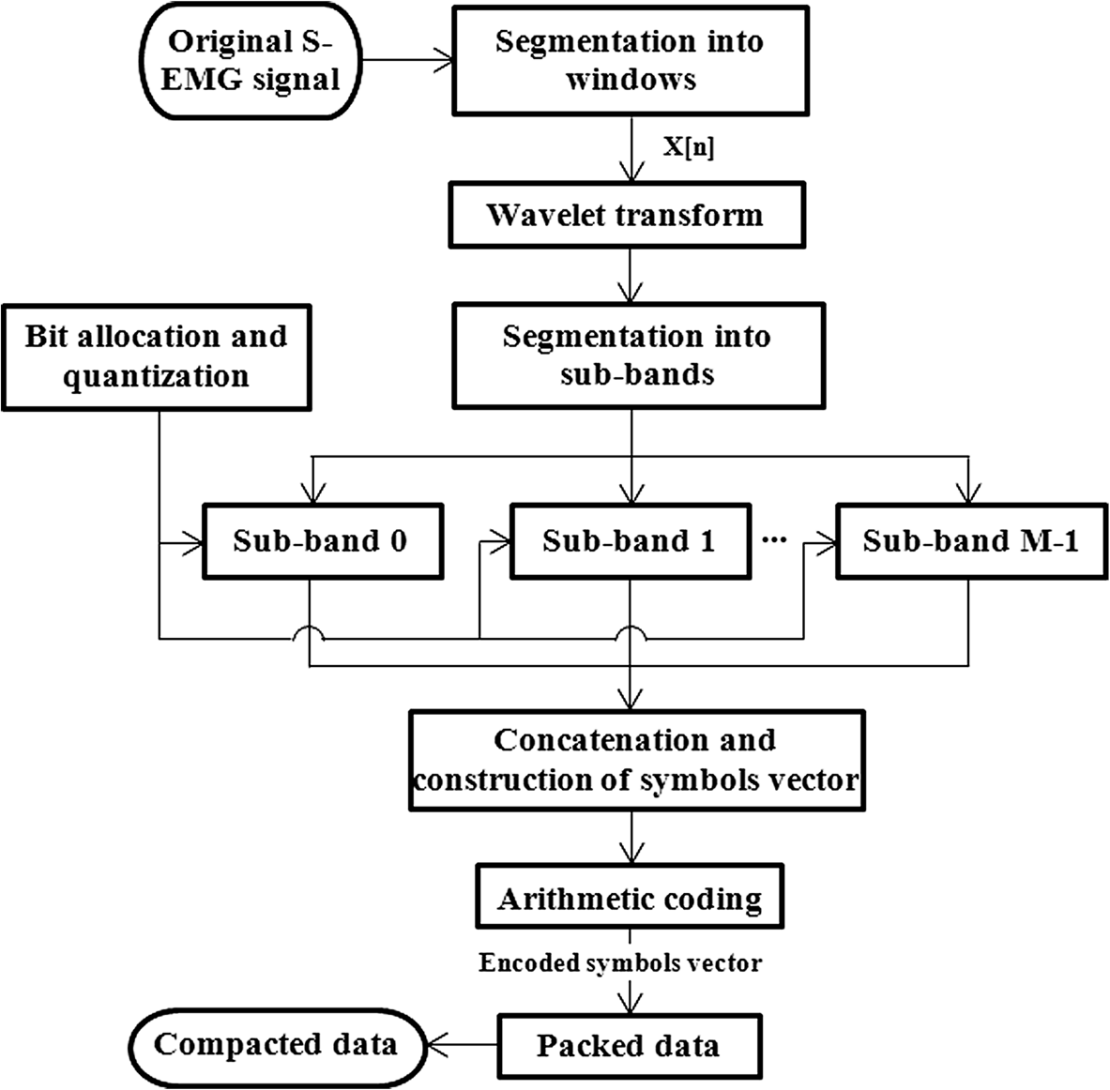
Block diagram of the S-EMG signal encoder.

Figure 1 illustrates, in a block diagram, the various modules that make up the S-EMG compression algorithm. *x*[*n*] is correspondent to the time domain signal while *X*[*k*] corresponds to the wavelet domain and *N* denotes the length of the sample window. The purpose of the orthogonal transform is to concentrate the energy of the signal into a smaller quantity of transform coefficients leading to a sparse representation in the transformed domain. The wavelet transform is implemented in an orthonormalized manner. This means that the dynamic range of the coefficients *X*[*k*] in the transformed space is less than or equal to the dynamic range of the signal in the time domain, meaning that the ratio of Equation (1) is met.

(1) $$\text{max}{\left\{X\left[k\right]\right\}}_{k=0,1,\ldots ,N-1}\leq 2^{R-1}$$

where *N* also corresponds to the length of the transformed coefficients vector and *R* to the word length (in bits) with which the sequence *x*[*n*] is digitized using fixed point representation. In the cases simulated in this study *N* = 2048 and all of the signals were digitized with a digital word length of *R* = 16 bits.

As already commented, the transform coefficient vector is also segmented, creating a total of *M* sub-bands. In the examples presented in this study *M* = 16 was used. Therefore, in each sub-band we have *N/M* transformed coefficients. There are *N/M* = 128 transformed coefficients in the simulation results presented in this paper.

The *N* transformed coefficients *X*[*k*], *k* = 0, 1, … , *N*–1, are quantized in each of the *M* sub-bands according to the relationship:

(2) $$X_{q}\left[k\right]=\mathrm{int}\left\{\frac{X\left[k\right]}{2^{R-1}}\lambda_{m}\right\}$$

The *N/M* coefficients pertaining to the same sub-band are, in principle, suitable for representation with the same digital word length. For a given *M* sub-band there are *N/M* wavelet transform coefficients associated, according to the mathematical relationship:

(3) $$k=\frac{N}{M}m,\frac{N}{M}m+1,\ldots ,\frac{N}{M}m+\frac{N}{M}-1\;\mathrm{where}\;m=0,1,\ldots ,M-1$$

The parameter *λ*_*m*_ is obtained for each of the *M* sub-bands from the estimators of the spectral shape which seek to model the spectral energy contour from the S-EMG signal considering that although the information is not stationary, it has a low-pass characteristic (described in the following section is how the *λ*_*m*_ scale factor of Equation 2 is obtained). Thus, as the index associated with the transformed coefficient increases, the smaller digital word length will be the indicated to quantize the coefficients of the respective sub-bands.

After finishing this step, the sub-band quantized coefficients are regrouped in order to construct a vector of symbols that represent a signal window of *N* sample length. The vector of symbols is then compressed by an entropy coder. Arithmetic coding [27] has been shown to be more efficient when compared to run-length and Huffman techniques [14]. In the last step, the data are packed generating a final representation of the S-EMG file.

### Spectral shape in dynamic bit allocation

Four models were studied and implemented for approximating the spectral shape: (1) Decreasing Linear Bit Allocation shape (DLA); (2) Decreasing Square-Root shape (DSR); (3) Decreasing Exponential shape (DEA) and (4) Rotated Hyperbolic Tangent shape (RHT). The curves are decreasing in magnitude and within them are the lengths of the digital words indicated by the numeric representation of each wavelet coefficient in each sub-band of the transformed vector.

In the following, the mathematical formalism associated with the spectral shape models proposed in this paper is presented. Vector *B*[*m*] stores the appropriate number of bits for each coefficient of the sub-band *m.* Parameter *m* indicates the index of the sub-band and M the amount of sub-bands used in the segmented spectrum. *Q* and *L* correspond to the longest and shortest digital word length indicated for coding the transformed coefficients vector.

### Decreasing linear bit allocation shape (DLA)

The spectral shape model is described with a decreasing linear curve that varies between the *Q* and *L* parameters as shown in Equation 4

(4) $$B\left[m\right]=\text{intsup}\left\{Q-\frac{Q-L}{M-1}m\right\}$$

### Decreasing square-root shape (DSR)

In this case, the model developed for approximating the spectral shape in the transformed space has amplitude decay in proportion to the square-root between the *Q* and *L* values as follows

(5) $$B\left[m\right]=\text{intsup}\left\{\xi \sqrt{C-m}\right\}$$

(6) $$\xi =\frac{Q}{\sqrt{C}}=\frac{L}{\sqrt{C+1-M}}$$

and

(7) $$C=\frac{Q^{2}\left(1-M\right)}{L^{2}-Q^{2}}$$

### Decreasing exponential shape (DEA)

In the decreasing exponential shape the number of bits indicated in *B*[*m*] decays exponentially from *Q* to *L*. This mathematically leads to the expressions below:

(8) $$B\left[m\right]=\mathrm{intsup}\left\{{\left(\frac{1}{b}\right)}^{m-p}\right\},b>1$$

(9) $$b=\sqrt[{p}]{Q}$$

(10) $$p=\frac{1-M}{\frac{\mathrm{lo}g_{10}\left(L\right)}{\mathrm{lo}g_{10}\left(Q\right)}-1}$$

### Rotated hyperbolic tangent (RHT)

**Figure 2:**
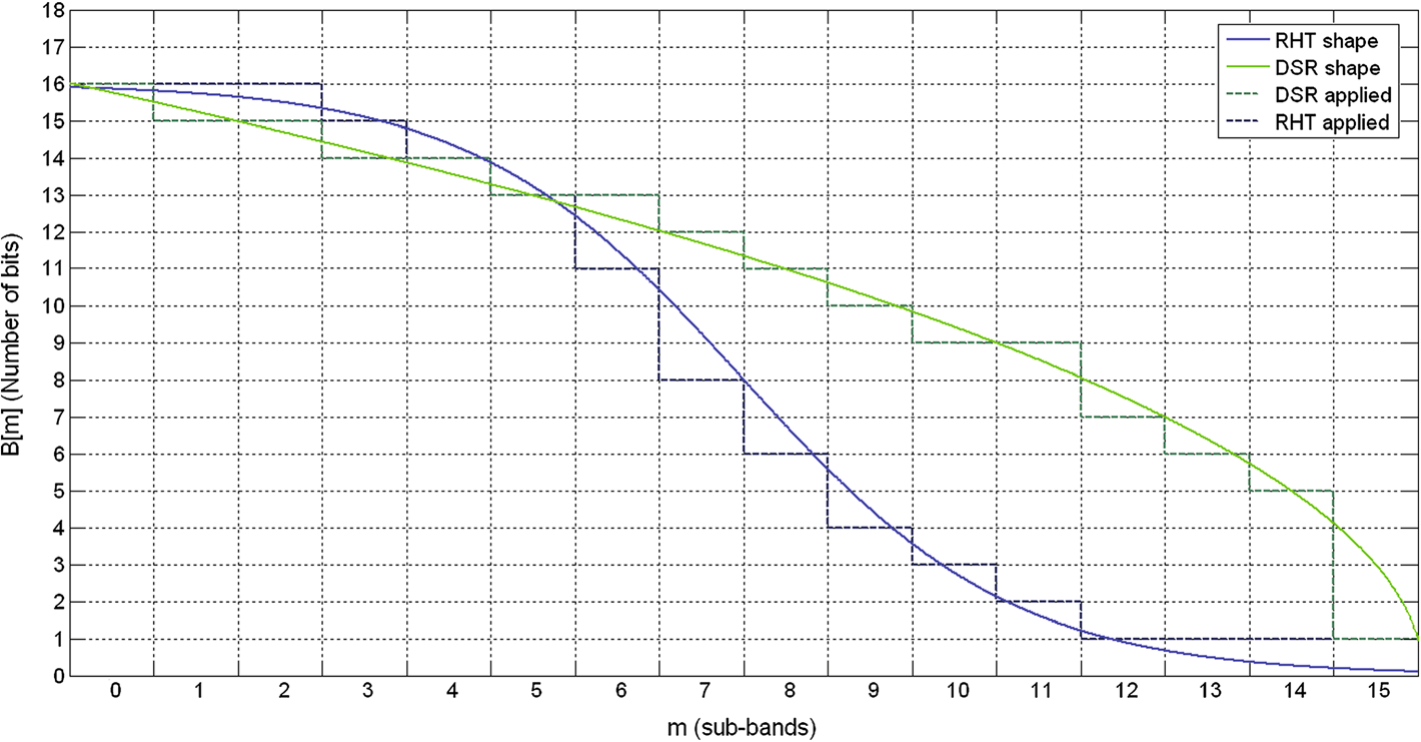
**Illustrative examples.** Decreasing Square-Root shape (DSR) and Rotated Hyperbolic Tangent Bit Allocation shape (RHT) models. The horizontal axis indicates the respective frequency bands and the vertical axis the number of bits associated with each frequency band. Smooth behavior of mathematical curves models can be viewed in these examples drawn in continuous lines. Shown in the dashed lines are the amounts of bits associated with variable B[m]. Using Equations (2) and (12) the quantized coefficients can be obtained.

In this spectral shape model a curve based on a π/2 radian rotated hyperbolic tangent function is proposed as illustrated in Figure 2. The parameters *α* and *β* indicate the behavior in the transition between *Q* and *L* in addition to the linear displacement of the midpoint, as shown in Figure 2, respectively.

(11) $$B\left[m\right]=\mathrm{intsup}\left\{\frac{Q}{2}\left[1-\mathrm{htan}\left(\alpha \left(m-\frac{M}{\beta }\right)\right)\right]\right\}$$

Having obtained *B*[*m*], for *m* = 0, 1, … , *M*–1 sub-bands, the scale factor *λ*_*m*_ may be calculated by

(12) $$\lambda_{m}=2^{B\left[m\right]}$$

After calculating the *λ*_*m*_ parameter, the quantized coefficient vector *X*_*q*_[*k*], *k* = 0, 1, … , *N*–1, may be reached as shown in Equation 2.

### Decoding algorithm

**Figure 3:**
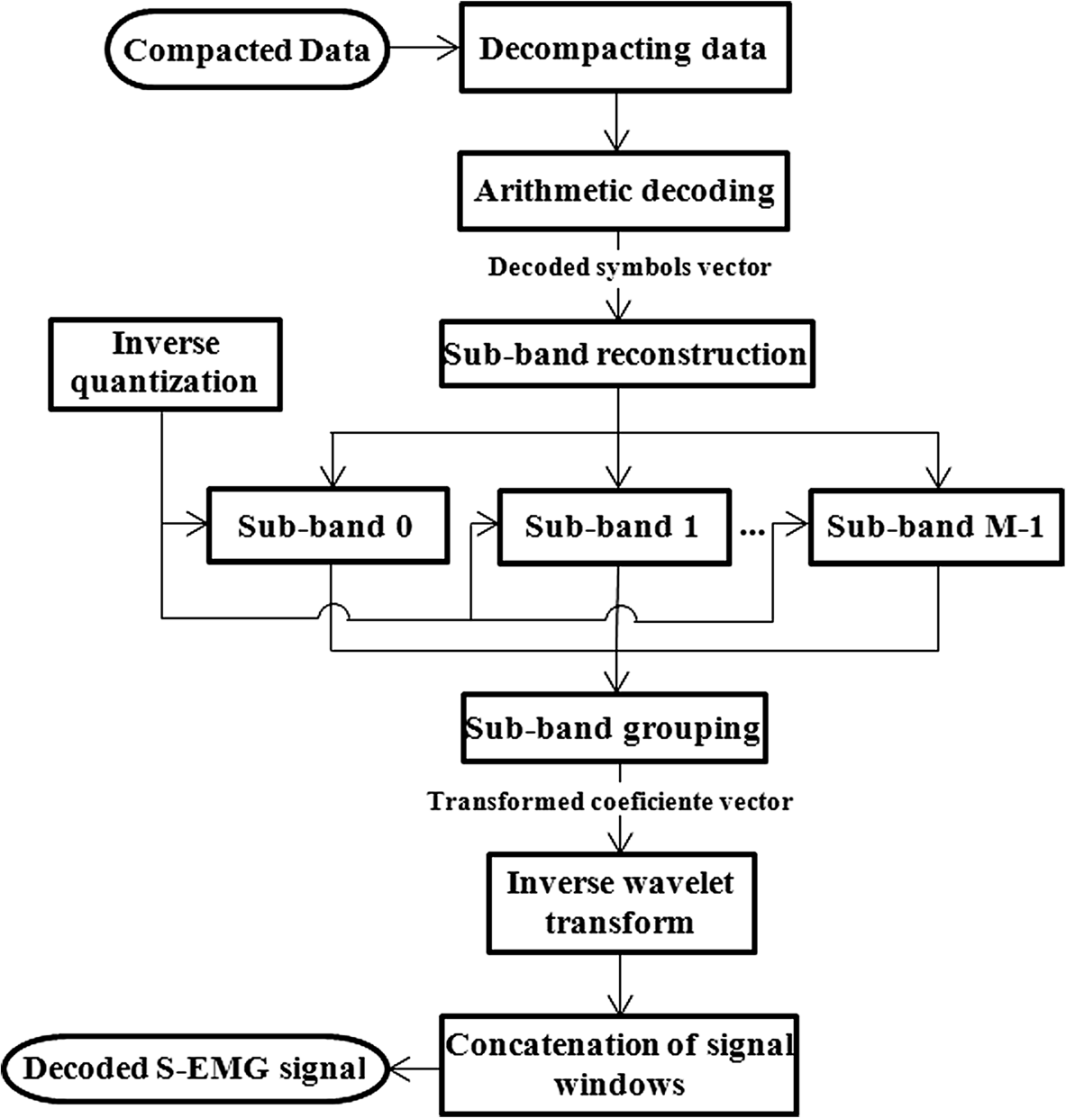
Block diagram of the S-EMG signal decoding.

Figure 3 shows the block diagram that describes the process for decoding the compressed data. First, the data is unpacked in order to have access to the information corresponding to each segment of the compressed signal. Next, entropy decoding is applied. The spectral sub-bands are reconstructed and in each sub-band, inverse quantization is carried out. The sub-bands are regrouped to obtain the coefficient vector to which an inverse wavelet transform is applied. As a result, a segment of *N* S-EMG signal samples is obtained. The various segments are concatenated in order to obtain the decoded signal.

Presented next are the metrics used to carry out the performance evaluation of the proposed algorithm.

### Metrics used for performance evaluation

The performance of the compression algorithm was objectively measured from two metrics: the compression factor (CF) and the percent residual difference (PRD). Currently, these criteria are the most commonly used in scientific literature [7,9,13-15,19-24] for evaluating the compression of electrophysiological signals. The compression factor is defined by (13),

(13) $$\mathrm{CF}=\frac{O_{S}-C_{S}}{O_{S}}\times 100\text{\%}$$

where *O*_*S*_ is the number of bits necessary for storing the original data and *C*_*S*_ is the amount of bits necessary for storing the compressed data.

The percent residual difference is defined in (14) as

(14) $$\mathrm{PRD}=\sqrt{\frac{\sum_{n=0}^{K-1}{\left(x\left[n\right]-\hat{x}\left[n\right]\right)}^{2}}{\sum_{n=0}^{K-1}x^{2}\left[n\right]}}\times 100\text{\%}$$

where *x*[*n*] is the original signal, $\hat{x}$[*n*] is the reconstructed signal and *K* is the total length in samples of the S-EMG signal.

To implement the orthogonal transform, the Daubechies-4 base with 8 levels of resolution was chosen. This choice was established based on a previous study having the de-correlation of the S-EMG signal in the transformed space and measurements of the capacity for compressing signal energy into fewer transformed coefficients as performance parameters [3].

### Experimental protocols used to evaluate performance

Two distinct experimental protocols were addressed in this research for evaluating the performance of the data compression technique proposed: (1) isometric protocol and (2) dynamic protocol.

### Proposed experimental isometric protocol

Pre-amplified surface electrodes (model *DE-02*, *DelSys Inc. Boston MA, USA*) were used in the S-EMG signals acquired in the experimental isometric protocol. The electrodes were positioned in order to get signals coming from the *biceps brachii* muscle. In all, 14 subjects were evaluated who underwent isometric stress force, maintaining 60% of their maximum voluntary contraction. The signals were fed into a data acquisition card with *LabVIEW (NI-DAQ for Windows, National Instruments*, USA). All signals were sampled at 2 kHz and digitized with 2 bytes/sample. The duration of the signals varies from 3 to 6 minutes.

### Proposed dynamic experimental protocol

When evaluating the proposed techniques with dynamic experimental protocol a set of S-EMG signals collected from the *vastus lateralis* muscle was used from 14 individuals riding a cycling simulator (Cateye CS1000, USA). In the experiment, pre-amplified surface electrodes were used (model DE-02, DelSys Inc. Boston MA, USA). The signals were fed into a data acquisition card with LabVIEW (NI-DAQ for Windows, National Instruments, USA). All signals were sampled at 2 kHz and quantized with 16 bits. The duration of the signals varies from 3 to 6 minutes.

In the next section, an evaluation of the proposed algorithm and performance comparison with other techniques found in scientific literature are also presented. Simulated results were obtained with the real S-EMG signal data bank.

## Results

**Figure 4:**
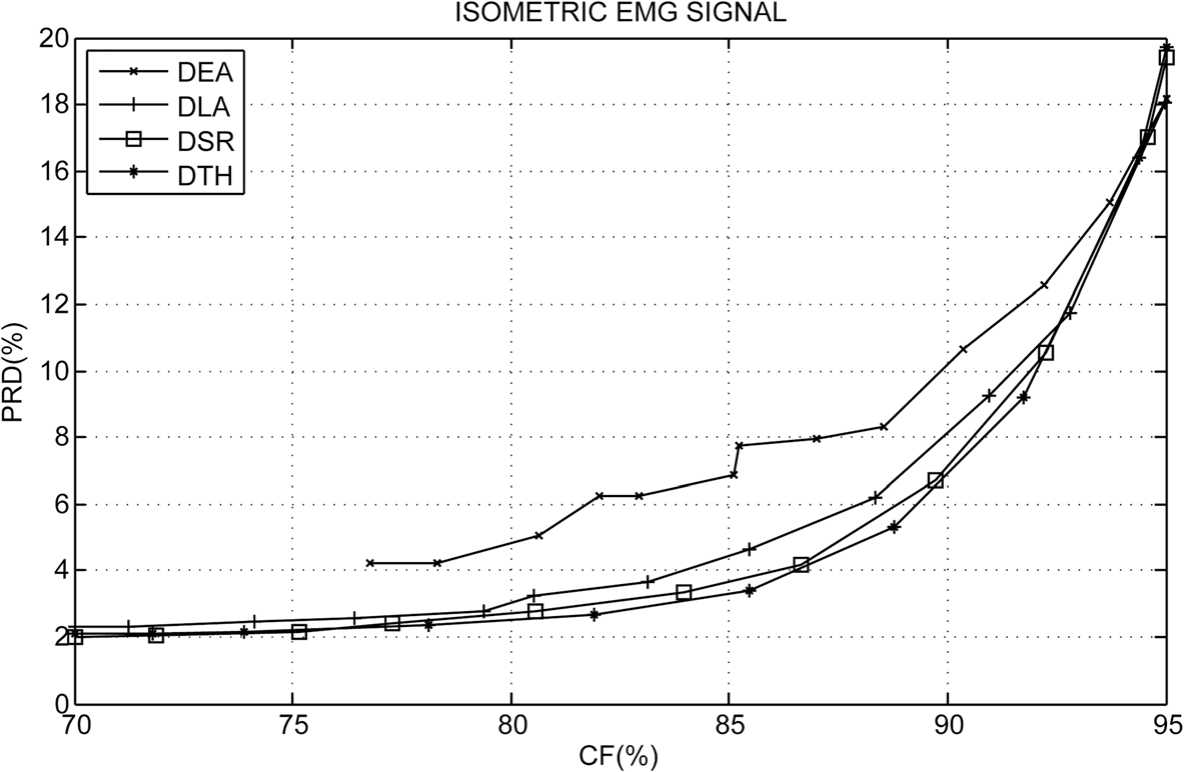
**Simulation results for isometric protocol.** Comparison of the proposed algorithm’s performance in the four spectral shapes implemented for the experimental isometric protocol, where: DLA–Decreasing Linear Bit Allocation Shape; RHT–Rotated Hyperbolic Tangent Bit Allocation Shape; DEA–Decreasing Exponential Bit Allocation Shape; and DSR–Decreasing Square-Root Bit Allocation Shape.

**Figure 5:**
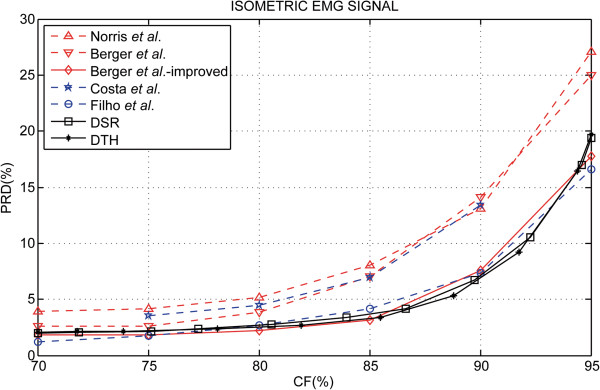
**Simulation results for isometric protocol.** Performance evaluation of the proposed method of S-EMG data compression: emphasized in this figure is the Decreasing Square-Root Bit Allocation Shape (DSR) and Rotated Hyperbolic Tangent Bit Allocation Shape (RHT) spectral shape approximation. Also shown in this illustrative scenery is a comparison of the performance evaluation with relevant works found in scientific literature: Norris *et al.*[7]–based on EZW (embedded zero-tree wavelets) scheme, Berger *et al.*[13]–based Wavelet Transform, neural network bit allocation procedure and Huffman entropy coding, Berger *et al.*–improved [14]–based Wavelet Transform, neural network bit allocation procedure and arithmetic entropy coding, Costa *et al.*[20]–based on two-dimensional technique to S-EMG compression, and Filho *et al.*[15]–based on recurrent patterns algorithm. It is important to notice that [13-15] and [20] used the same signal data bank used in this work.

**Figure 6:**
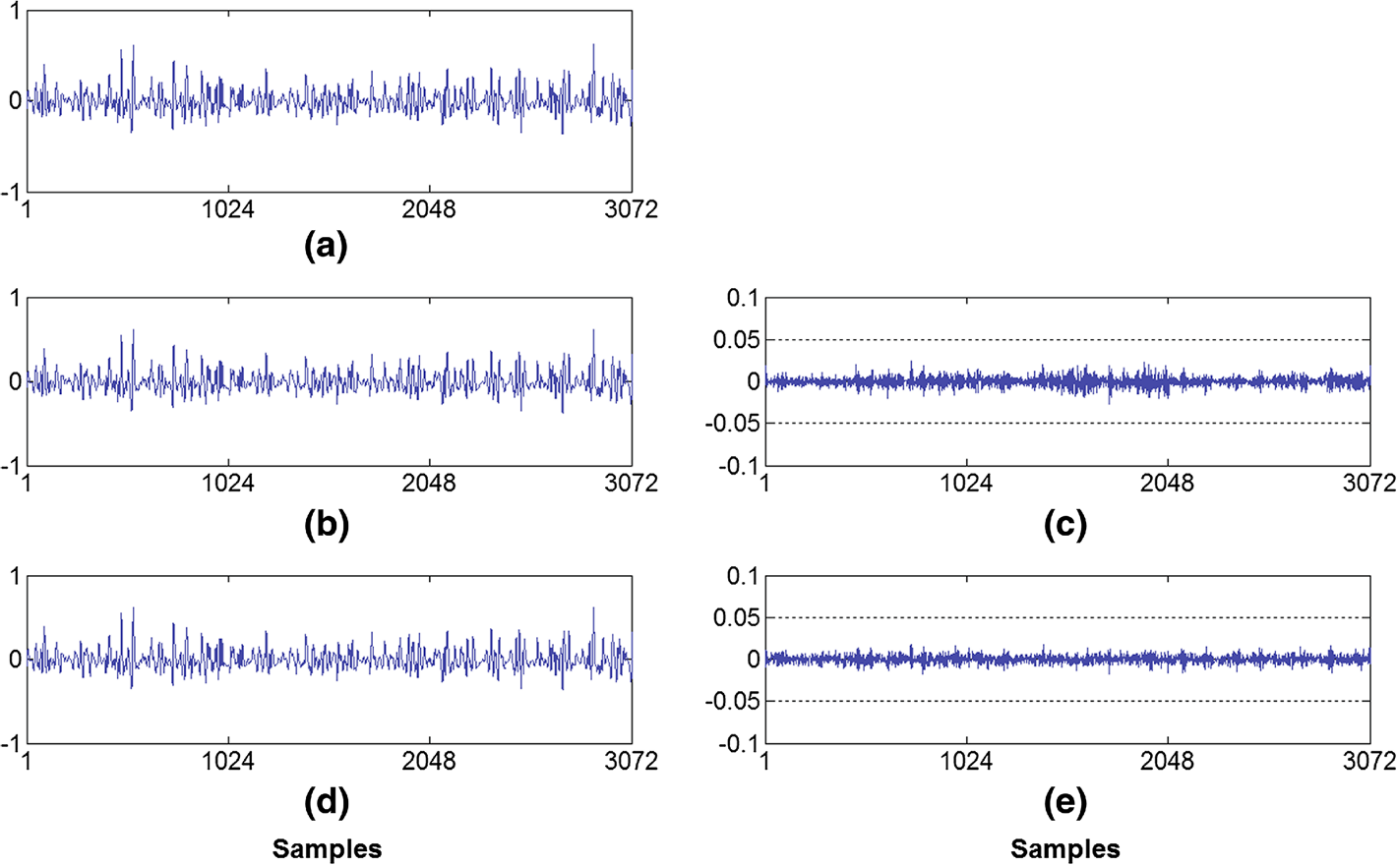
**Qualitative examples. (a)** the original signal with a 3072 sample window, **(b)** the same window of the signal reconstructed using the DLA bit allocation shape, **(c)** the difference between the original and decoded signal for the DLA bit allocation shape (with CF = 85.86% and PRD = 4.67%), **(d)** the decoded signal using the RHT bit allocation shape (with CF = 85.92% and PRD = 3.44%), and **(e)** the difference between the original and decoded signal for the RHT bit allocation shape.

Figure 4 shows a summary of the PRD results according to the CF for the S-EMG signal data bank used for isometric experimental protocol. In Figure 5, a comparison of the proposed algorithm using the rotated hyperbolic tangent bit allocation shape (RHT) and the decreasing square-root bit allocation shape (DSR) with other results reported in scientific literature can be observed. Table 1 succinctly illustrates the difference in performance between the coders analyzed and Figure 6 shows examples of segments of the original signal, of the decoded signal and of the error signal obtained (difference between the signals).

**Table 1:** Isometric–performance evaluation of the coders–PRD (%)

	**Compression factor‒CF (%)**					
	**70**	**75**	**80**	**85**	**90**	**95**
Norris *et al*. [7]	3.90	4.12	5.20	8.02	13.08	27.10
Berger *et al*. [13]	2.57	2.63	3.85	7.01	14.14	24.95
Berger *et al.*-improved [14]	1.79	1.80	**2.24**	**3.13**	7.61	17.76
Filho *et al.*[15]	**1.21**	**1.75**	2.64	4.18	7.33	**16.61**
DEA	–	–	4.82	6.83	10.17	18.17
DLA	2.30	2.49	3.01	4.42	8.13	18.30
DSR	2.00	2.15	2.69	3.68	7.09	19.40
RHT	2.07	2.22	2.52	3.31	**6.88**	19.74

Best results in bold, i.e., lowest PRD for a given CF (arranged column-wise).

**Figure 7:**
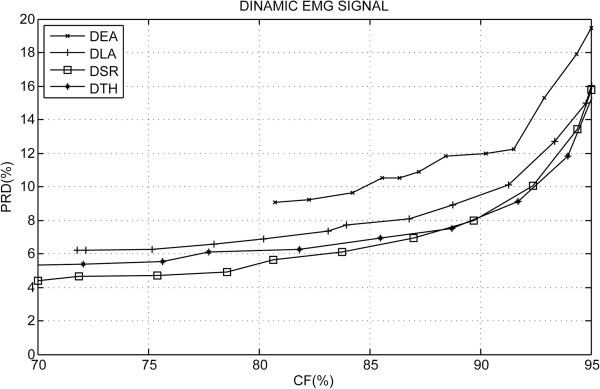
**Simulation results for dynamic protocol.** Performance comparison of the proposed algorithm for the four spectral shapes implemented in the dynamic experimental protocol, where: DLA–Decreasing Linear Bit Allocation Shape; RHT–Rotated Hyperbolic Tangent Bit Allocation Shape; DEA–Decreasing Exponential Bit Allocation Shape and DSR–Decreasing Square-Root Bit Allocation Shape.

**Figure 8:**
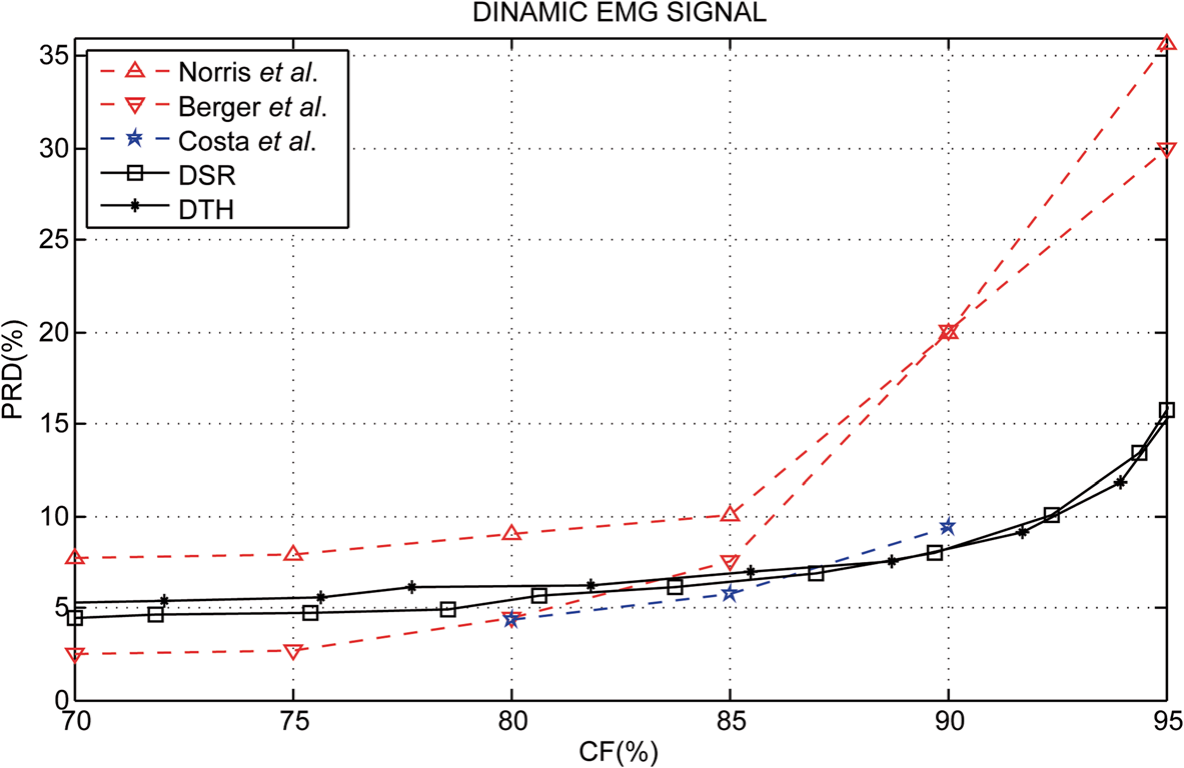
**Simulation results for dynamic protocol.** Performance comparisons of the proposed method of S-EMG data compression in the studies reported in scientific literature. In this figure, results are shown which were obtained using the decreasing square-root (DSR) and Rotated Hyperbolic Tangent Bit Allocation Shape (RHT) spectral shape approximation. Also, this figure shows performance results found in Norris *et al.*[7]–based on the EZW (embedded zero-tree wavelets) scheme, Berger *et al.*[13]–based Wavelet Transform, neural network bit allocation procedure and Huffman entropy coding, and Costa *et al.*[20]–based on a two-dimensional technique for S-EMG compression. It is also important to notice that [13] and [20] used the same dynamic S-EMG signal data bank used in this work.

**Figure 9:**
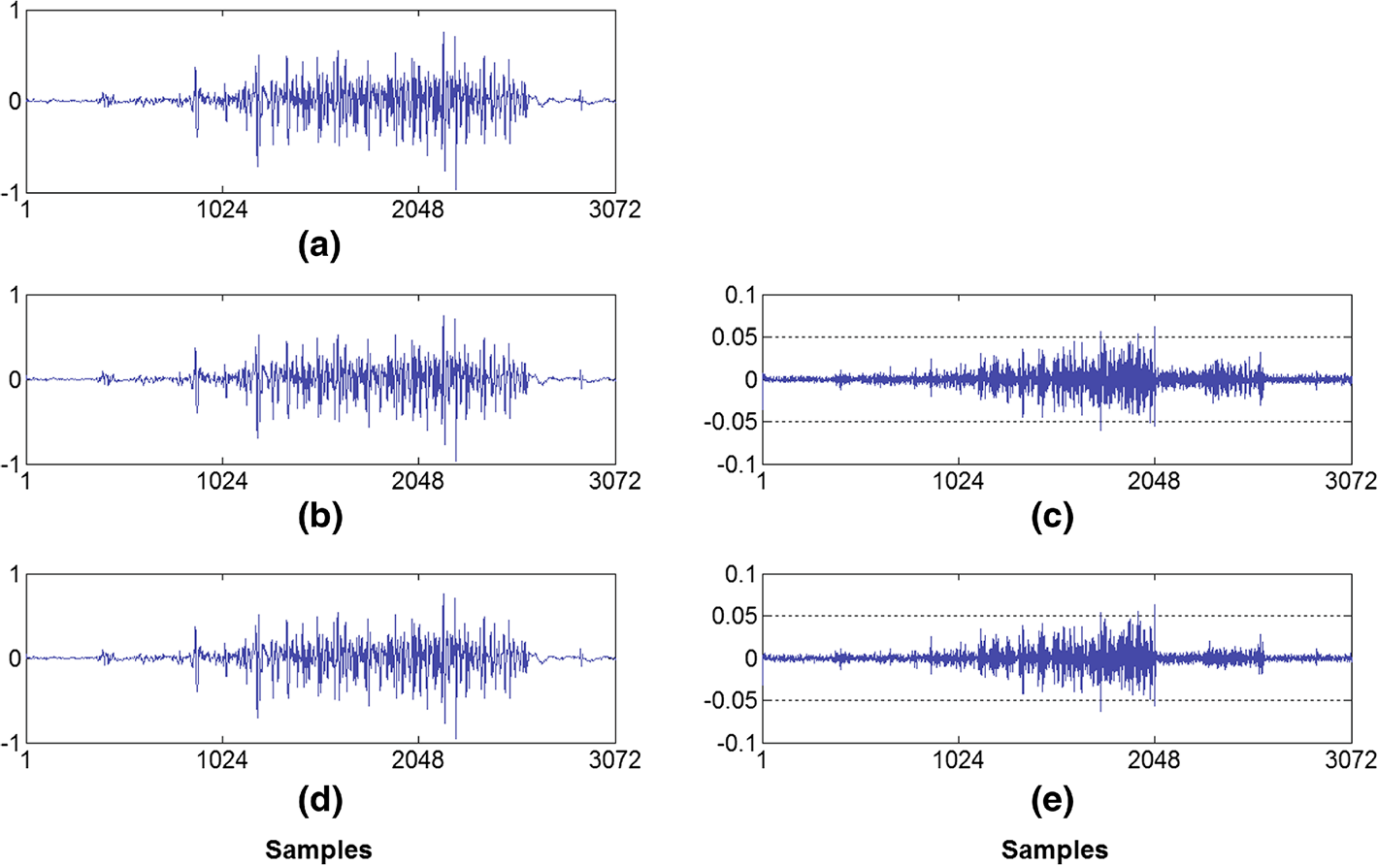
**Reconstruction examples using the different spectral shapes. (a)** window with 3072 original S-EMG signal samples; **(b)** signal reconstruction with DSR shape (CF = 88.54% and PRD = 8.93%), **(c)** DSR reconstruction error, **(d)** signal reconstructed with DLA (CF = 87,41% and PRD = 9.92%), **(e)** DLA reconstruction error.

Results for dynamic experimental protocol are presented in Figure 7 and it shows a summary of the PRD behavior according to the CF for the S-EMG signal bank. In Figure 8 a performance comparison may be observed with other algorithms published in scientific literature. Table 2 summarizes the results found of the percent residual difference (PRD) for specific values of the compression factor (CF). Figure 9 shows a segment of the original signal (a), two examples of the signals reconstructed using the proposed algorithm in (c) and (e) and the respective signal error obtained (difference between the original and reconstructed signal).

**Table 2:** Dynamic–performance evaluation of the coders‒PRD (%)

	**Compression factor‒CF (%)**					
	**70**	**75**	**80**	**85**	**90**	**95**
Norris *et al.*[7]	7.75	7.93	9.06	10.02	19.98	35.71
Berger *et al.*[13]	**2.44**	**2.70**	4.41	7.52	20.10	29.96
Costa *et al.*[20]	–	–	**4.39**	**5.77**	9.39	–
DEA	–	–	–	10.13	11.94	19.44
DLA	–	6.23	6.84	7.86	9.50	16.05
DSR	4.41	4.70	5.41	6.40	**8.22**	**15.76**
RHT	5.46	6.15	6.59	7.44	10.23	16.24

Best results in bold, i.e., lowest PRD for a given CF (arranged column-wise).

## Discussions

The algorithm evaluations with isometric and dynamic experimental protocols were implemented. Ahead, the performances of two distinct experimental protocols addressed in this research are discussed.

### Performance analysis for S-EMG isometric experimental protocol

For isometric protocol, the PRD results according to the CF is illustrate in Figure 4. In this figure it can be observed that the decreasing exponential spectral shape’s performance is inferior to the other spectral shape models implemented. This difference in performance is more significant for a smaller CF. On the other hand, for isometric S-EMG signal compression, the rotated hyperbolic tangent spectral shape model performed slightly superior to the others proposed. Insofar as the CF grows, the performance curves approach each other.

A comparison of the proposed algorithm using the rotated hyperbolic tangent bit allocation shape (RHT) and the decreasing square-root bit allocation shape (DSR) with other results reported in scientific literature can be observed in Figure 5. In this figure may be verified that the performance curves presented by Filho *et al.*[15], Berger *et al.*–improved [14] and through the proposed technique using the spectral shapes RHT and DSR are similar. Table 1 succinctly illustrates the difference in performance between the coders analyzed. Highlighted in the table are the lower PRD values for the CF values listed. For 70%, 75% and 95% CF the results reported by Filho *et al.*[15] have the lowest PRD values. 80% and 85% CF Berger *et al.*–improved [14] had a performance slightly superior to other techniques and for 90% CF the compression technique presented in this paper using the RHT spectral shape model had the lowest PRD value.

Figure 6 shows examples of segments of the original signal, of the decoded signal and of the error signal obtained (difference between the signals). In the example, the waveforms have been normalized for the purpose of illustration. Figure 6b was obtained by using the decreasing linear bit allocation shape. In turn, Figure 6d was reached when using the rotated hyperbolic tangent shape. In both examples, a compression ratio of approximately 85% was sought.

### Performance analysis for S-EMG dynamic experimental protocol

Figure 7 shows a summary of the PRD results according to the CF for the S-EMG signal bank with dynamic experimental protocol. The S-EMG compression algorithm was implemented for each of the proposed spectral shape models. Analogous to the isometric case, the spectral shape model with the worst performance was DEA. However, unlike the isometric case, the DSR spectral shape obtained a performance slightly better than the RHT.

In Figure 8, a performance comparison may be observed with other algorithms published in scientific literature. It should be noted that Berger *et al.*–improved [14] and Filho *et al.*[15] did not present results for dynamic experimental protocol S-EMG signal compression. Table 2 summarizes the results found of the percent residual difference (PRD) for specific values of the compression factor (CF). For 70% and 75% CF the results reported by Berger *et al.*[13] have the lowest PRD values. For 80% and 85% CF Costa *et al.*[20] show better performance when compared to the other techniques listed in Table 2. For 90% and 95% CF the results of the compression techniques presented in this study using the DSR spectral shape model have the lowest PRD values.

Figure 9 shows a segment of the original signal (a), two examples of the signals reconstructed using the proposed algorithm in (c) and (e) and the respective signal error obtained (difference between the original and reconstructed signal). The results for the decreasing square-root shape model (DSR) and the decreasing linear bit allocation shape (DLA) may be observed in this example.

## Conclusions

The S-EMG signal compression algorithm described in this study revealed itself to be very efficient. The proposed dynamic bit allocation scheme for transformed coefficients based on a spectral shape model integrated with a quantization process and entropy coding leads to a high accuracy of S-EMG waveforms coding. It also allows greater liberty for adjusting the spectral content to the length of the digital word to be used in the representation of transformed wavelets coefficients.

For isometric S-EMG experimental protocol the rotated hyperbolic tangent spectral shape model (RHT) performed slightly superior to the others proposed models. However, in dynamic S-EMG experimental protocol, it was observed for similar conditions to the compression factor (CF) the decreasing square-root spectral shape model (DSR) has a percent residual difference slightly lower than others bit allocation spectral shape model.

Dynamic experimental protocol has intervals with and without muscle activation. The electrophysiological behavior and large dynamic range variation in the S-EMG intervals with and without muscle activation lead to different non-stationary power concentration in spectral sub-bands when it is compared with isometric protocol. As a result, different CF *x* PRD performances are obtained for isometric and dynamic experimental protocols for the various proposed models of spectral shapes.

An improvement in performance with respect to the objective evaluation metrics can be investigated through the development of new models of spectral shapes. Another approach for refining the technique is envisioned by local adaptation of the spectral shape model.

## Competing interests

MHT, MVCC and FAON have no competing interests.

## Authors’ contributions

MHT and MVCC worked with algorithm implementation, improvement and evaluation. FAON is the Ph.D. thesis advisor of MHT and MVCC. He worked with the algorithm design and evaluation. All authors read and approved the final manuscript.
